# Effect of traditional plants in Sri Lanka on skin fibroblast cell number

**DOI:** 10.1016/j.dib.2018.05.019

**Published:** 2018-05-16

**Authors:** Katsura Sano, Takao Someya, Kotaro Hara, Yoshimasa Sagane, Toshihiro Watanabe, R.G.S. Wijesekara

**Affiliations:** a ALBION Co. Ltd., 1-7-10 Ginza, Chuo-ku, Tokyo 104-0061, Japan; b Department of food and Cosmetic Science, Faculty of Bioindustry, Tokyo University of Agriculture, 196 Yasaka, Abashiri, Hokkaido 099-2493, Japan; c Department of Aquaculture and Fisheries, Faculty of Livestock, Fisheries and Nutrition, Wayamba University of Sri Lanka, Makandura, Gonawila 60170, Sri Lanka

**Keywords:** Cell number, Fibroblasts, Calcein assay, Traditional plant, Medical herb

## Abstract

This article describes the effects of extracts of several plants collected in Sri Lanka on the cell number of human skin fibroblasts. This study especially focuses on the plants traditionally used in indigenous systems of medicine in Sri Lanka, such as Ayurveda, as described below (English name, “local name in Sri Lanka,” scientific name). Bougainvillea plant, “bouganvilla,” *Bougainvillea grabla* (Nature׳s Beauty Creations Ltd., 2014) [1], purple fruited pea eggplant,”welthibbatu,” *Solanum trilobatum* (Nature׳s Beauty Creations Ltd., 2014) [2], country borage plant, “kapparawalliya,” *Plectranthus amboinicus* (Nature׳s Beauty Creations Ltd., 2014) [3], malabar nut plant, “adhatoda,” *Justicia adhatoda* (Nature׳s Beauty Creations Ltd., 2014) [4], long pepper plant,”thippili,” *Piper longum* (Nature׳s Beauty Creations Ltd., 2014) [5], holy basil plant, “maduruthala,” *Ocimum tenuiflorum* (Nature׳s Beauty Creations Ltd., 2014) [6], air plant, “akkapana,” *Kalanchoe pinnata* (Nature׳s Beauty Creations Ltd., 2014) [7], plumed cockscomb plant, “kiri-henda,” *Celosia argentea* (Nature׳s Beauty Creations Ltd., 2014) [8], neem plant,”kohomba,” *Azadirachta indica* (Nature׳s Beauty Creations Ltd., 2014) [9], emblic myrobalan plant, “nelli,” *Phyllanthus emblica* (Nature׳s Beauty Creations Ltd., 2014) [10]. Human skin fibroblast cells were treated with various concentration of plant extracts (0–3.0%), and the cell viability of cells were detected using calcein assay. The cell viabillity profiles are provided as line graphs.

**Specifications Table**

**Table:** 

Subject area	*Biology*
More specific subject area	*Cell biology*
Type of data	*Graph*
How data was acquired	*Fluorescent microscope (SpectraMax^®^ i3x, MOLECULAR DEVICES)*
Data format	*Analyzed*
Experimental factors	*Treatment of fibroblast cells with various concentrations of plant extracts*
Experimental features	*Analysis of cell number by calcein assay*
Data source location	*Negombo, Sri Lanka*
Data accessibility	*Data are available within this article*

**Value of the data**

• Data represent changes in fibroblast cell numbers after exposure to several plant extracts.
• These data indicate that several plant extracts regulate fibroblast cell numbers in the dermis, and could be further investigated as pharmacologic and cosmetic agents.
• This article investigates natural agents for the treatment of diseases of dermal tissues.

## 1 Data

This data article contains line graphs showing the effects of extracts of several plants harvested in Negombo, Sri Lanka, on the cell number of human skin fibroblasts. Cells were treated with various concentrations (0–3.0%) of each plant extracts for 24 h, and percent cell viability was calculated relative to that of untreated controls. Data represent the mean±SE values from triplicate independent experiments (*P<0.05, **P<0.001 and ***P<0.001 vs. control).

## 2 Experimental design, materials and methods

All plants were collected from a medicinal garden at the Institute of Traditional Plants in Sri Lanka (Negombo, Sri Lanka). Each plants were cleaned with water, dried under shade conditions for 3-days, and cutted in pieces with scissors. Each plant extract were extracted with specific solvents at room temperature for 24 h as described below (plant name, part, solvent(ratio)). The extract were filtered through a filter paper and 0.22 µm filter [11].

Bougainvillea plant, “bouganvilla,” *Bougainvillea grabla* [1], [12], flower, 70% EtOH(7.5 times(w/w)); purple fruited pea eggplant,”welthibbatu,” *Solanum trilobatum* [2], [13], shoot, 70% EtOH(4.0 times(w/w)); country borage plant, “kapparawalliya,” *Plectranthus amboinicus* [3], [14], leaf,70% EtOH(3.0 times(w/w)); malabar nut plant, “adhatoda,” *Justicia adhatoda* [4], [15], leaf, 70%(5.0 times(w/w)) EtOH; long pepper plant,”thippili,” *Piper longum* [5], [16], leaf, 70% EtOH(7.5 times(w/w)); holy basil plant, “maduruthala,” *Ocimum tenuiflorum* [6], [17], shoot, 70% EtOH(3.0 times(w/w)); air plant, “akkapana,” *Kalanchoe pinnata* [7], [18], leaf, 70% EtOH(2.0 times(w/w)); plumed cockscomb plant, “kiri-henda,” *Celosia argentea* [8], [19], shoot, 70% EtOH(2.5 times(w/w)); neem plant,”kohomba,” *Azadirachta indica* [9], [20], leaf, 50% BG(15.0 times(w/w)); emblic myrobalan plant, “nelli,” *Phyllanthus emblica* [10], [21], leaf, 70% EtOH(7.5 times(w/w)).

## 3 Fibroblast cell culture

Normal human dermal skin fibroblasts, RIKEN original (NB1RGB), were provided by the RIKEN BRC through the National Bio-Resource Project of the MEXT, Japan. The cells were cultured in Minimum Essential Media-alpha (MEMα; Life Technologies Corp.) supplemented with 10% fetal bovine serum (FBS; Biowest) and 0.2% NaHCO_3_. Cells were grown at 37 °C in a humidified incubator containing 5% CO_2,_ according to the manufacturer׳s instructions. For all of the experiments, human fibroblasts were seeded (3×10^3^ cells/well) in a 96-well plate, and incubated for 8 h with culture media containing 10% FBS. The cells were subsequently subjected to serum starvation for 16 h with serum-free MEMα [22], [23].

## 4 Cell number analysis (calcein assay)

To determine cell viability, cells were seeded (3×10^3^ cells/well) in a 96-well plate. Cells were exposed to various concentrations of plant extracts for 24 h. The cells were then stained with 10 mM calcein-AM (Dojindo) in the dark for 30 min at 37 °C and washed with phosphate-buffered saline (PBS). The fluorescence intensity (em/ex, 485/530 nm) of each well was measured using a SpectraMax® i3x fluorescence microplate reader (MOLECULAR DEVICES). Data were calculated as the percent cell viability compared to that of controls without plants extracts treatment and have been presented as the mean and SE values for triplicate wells.

## 5 Statistical analysis

**Fig. 1:**
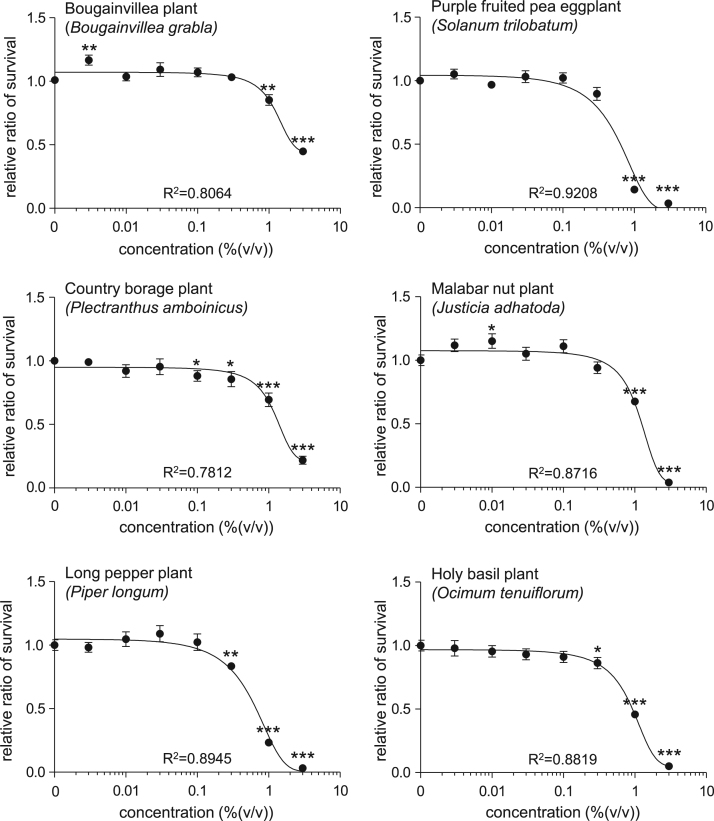

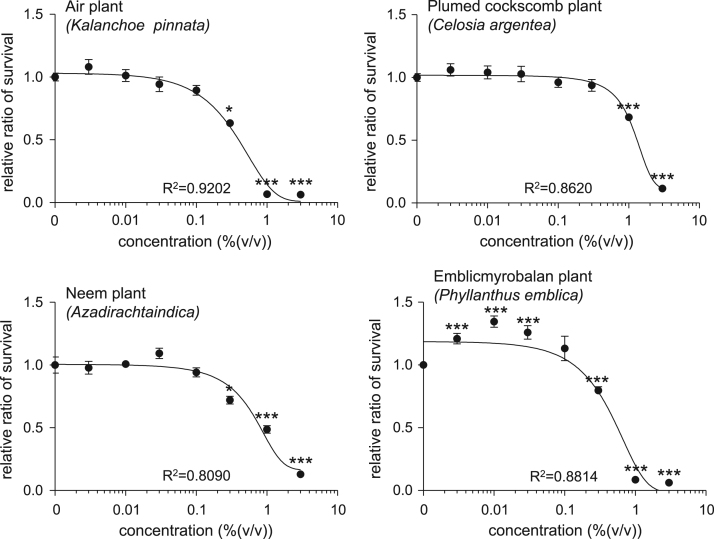
Cell viability of NB1RGB detected by calcein assay. Cells were treated with various concentrations (0–3.0%) of each plant extracts for 24 h, and percent cell viability was calculated relative to that of untreated controls. The values are shown as the mean±SE of three independent experiments.

All the values have been reported in terms of mean±SE values. The data were analyzed using the Student׳s *t*-test. A *P* value less than 0.05 was considered to be statistically significant (Fig. 1).
